# Molecular Classification of Endometrial Cancer in the Era of Precision Oncology: Prognostic Significance, Emerging Biomarkers, and Personalized Therapeutic Strategies—A Systematic Review

**DOI:** 10.3390/diagnostics16182902

**Published:** 2026-09-09

**Authors:** Gulinur Chinaliyeva, Azat Chinaliyev, Zhanna Amirbekova, Amankeldi Salybekov, Aida Shakirova, Didar Khassenov, Zhandos Burkitbayev, Nino Dznelashvili

**Affiliations:** 1 Department of Doctoral Studies, Karaganda Medical University, Karaganda 100008, Kazakhstan; 2 National Research Oncology Center, Astana 010000, Kazakhstan; medicinaastana@gmail.com (A.C.);; 3 Department of Obstetrics, Gynecology and Perinatology, Karaganda Medical University, Karaganda 100008, Kazakhstan; 4 Qazaq Institute of Innovative Medicine, Astana 020000, Kazakhstan; 5 Women’s Health Department, National Research Oncology Center, Astana 010000, Kazakhstan; 6 Healthycore Israel-Georgian Multi-Profile Clinic, Tbilisi 0112, Georgia

**Keywords:** endometrial cancer, molecular classification, TCGA, ProMisE, precision oncology, biomarkers, POLE, mismatch repair deficiency, microsatellite instability, p53 immunohistochemistry, multiple-classifier tumors, NSMP

## Abstract

**Background/Objectives**: Molecular classification has reshaped risk assessment in endometrial cancer (EC), but the strength and clinical applicability of the evidence vary across molecular subgroups, assay platforms, patient populations, and treatment settings. This systematic review critically evaluated the prognostic and predictive value of TCGA-derived classifiers, additional biomarkers, multiple-classifier tumors, and molecularly informed therapeutic strategies. **Methods**: A protocol was developed a priori but was not prospectively registered. PubMed/MEDLINE, Embase, Scopus, Web of Science, and the Cochrane Library were searched for primary studies published from January 2013 through June 2026. Clinical guidelines were used only to provide clinical context and were not included in the formal evidence set. Because of substantial methodological heterogeneity, findings were synthesized narratively by study design and clinical question. Risk of bias was assessed using QUADAS-2, the Newcastle–Ottawa Scale, and RoB 2. **Results**: Seventy-four studies involving approximately 42,000 patients were included. POLE-mutated tumors showed the most favorable outcomes in most cohorts, whereas p53-abnormal tumors, defined by abnormal immunohistochemical patterns, generally had the poorest prognosis. Mismatch repair deficiency identified by immunohistochemistry and microsatellite instability-high status identified by PCR- or NGS-based testing were highly related but analytically distinct biomarkers and were not treated as exact synonyms. The NSMP group remained heterogeneous; integrated assessment of estrogen/progesterone receptor expression, CTNNB1, L1CAM, ARID1A, and chromosome 1q alterations may improve risk discrimination, although most of these markers remain investigational. In multiple-classifier tumors, available data support a practical hierarchy in which a pathogenic POLE mutation generally takes precedence, followed by MMR deficiency and then p53 abnormality. Across studies, molecular classification usually added prognostic information to clinicopathologic assessment, but the magnitude of benefit varied, and no overall certainty rating was assigned. **Conclusions**: Molecular classification is incorporated into contemporary guidelines and can support prognostic assessment and treatment selection when interpreted together with stage, histology, and other clinicopathologic factors. The strongest current clinical utility relates to POLE, MMR/MSI, p53 immunohistochemistry, and HER2 in selected settings; additional NSMP and multi-omics biomarkers require prospective validation, assay standardization, and demonstration of clinical utility before routine adoption.

## 1. Introduction

Endometrial cancer (EC) is the most common gynecologic malignancy in many high-income countries and represents a growing global health challenge. GLOBOCAN 2022 estimated more than 420,000 new cases and over 97,000 deaths from cancer of the uterine corpus worldwide [1].

Although incidence remains highest in North America, Northern Europe, and Australia, substantial increases have also been reported in many middle-income countries, reflecting population aging, urbanization, and the increasing prevalence of obesity and metabolic disorders [2,3].

The burden of EC is projected to increase further. Population-based estimates suggest that incidence and mortality may rise markedly over the coming decades if current trends persist [4]. Obesity is the strongest modifiable risk factor and may account for a substantial proportion of cases in high-income countries [5]. Other established risk factors include diabetes mellitus, hypertension, nulliparity, prolonged estrogen exposure, polycystic ovary syndrome, and hereditary cancer syndromes, particularly Lynch syndrome [6,7]. A clinically important minority of cases occurs in premenopausal and reproductive-age patients, underscoring the relevance of fertility-preserving strategies [8].

Historically, EC was classified primarily by histopathologic characteristics. The dualistic model proposed by Bokhman in 1983 separated estrogen-dependent type I tumors, usually low-grade endometrioid carcinomas, from estrogen-independent type II tumors, including serous and clear-cell carcinomas with more aggressive behavior [9]. Although clinically influential, this framework did not capture the molecular and outcome heterogeneity within each histologic category [10,11].

The limitations of morphology-based stratification are evident when tumors with similar histology and grade follow markedly different clinical courses. Histologic typing and grading also show interobserver variability, particularly among high-grade endometrioid, serous, and clear-cell carcinomas [12]. Reliance on clinicopathologic features alone may therefore contribute to both overtreatment and undertreatment [13].

A major conceptual shift occurred in 2013, when The Cancer Genome Atlas (TCGA) integrated genomic, transcriptomic, proteomic, and epigenomic data from 373 endometrial carcinomas and identified four molecular groups: POLE-ultramutated, microsatellite instability/hypermutated, copy-number-low, and copy-number-high tumors [14]. Clinically applicable surrogate classifiers subsequently translated these groups into POLE-mutated (POLEmut), mismatch repair-deficient (MMRd), no specific molecular profile (NSMP), and p53-abnormal (p53abn) categories [15,16,17].

Subsequent validation studies confirmed that TCGA-derived classifiers provide clinically relevant prognostic information across diverse cohorts, leading to practical algorithms such as ProMisE and TransPORTEC [15,16,17]. Similarly, p53abn refers to abnormal immunohistochemical patterns, whereas TP53 mutation denotes a genomic alteration; p53 immunohistochemistry is a useful surrogate, but occasional discordance and subclonal patterns occur [18,19]. Important assay distinctions nevertheless remain. MMRd is commonly defined by loss of one or more MMR proteins on immunohistochemistry, whereas MSI-H is detected by PCR- or NGS-based testing; the results are highly concordant but are not identical [20]. A small proportion of tumors show more than one classifying feature and require hierarchical interpretation [21]. Molecular information has been incorporated into contemporary ESGO/ESTRO/ESP and NCCN recommendations and into the FIGO 2023 staging framework [22,23,24].

In parallel, molecular oncology has expanded treatment options. MMRd or MSI-H status predicts sensitivity to immune checkpoint inhibition, HER2 can identify candidates for HER2-directed therapy in selected tumors, and pathogenic POLE mutations may inform de-escalation research [25,26,27]. Other alterations, including FGFR2 and PI3K-pathway abnormalities, are being evaluated in biomarker-selected trials.

Thus, molecular classification is best viewed not as a replacement for morphology and stage, but as an additional layer of biologic information that can refine prognosis and treatment selection.

Several challenges remain unresolved: the marked heterogeneity of NSMP tumors; the classification and management of multiple-classifier tumors; variable assay platforms, thresholds, and reporting standards; and the distinction between biomarkers that are guideline-integrated and those that remain investigational [20,21,28,29,30].

This systematic review therefore aimed to critically evaluate evidence on TCGA-derived molecular classification in EC, compare the prognostic and predictive value of individual subgroups, distinguish established from investigational biomarkers, examine multiple-classifier tumors, and assess implications for personalized treatment and clinical implementation.

## 2. Materials and Methods

### 2.1. Protocol and Registration

This systematic review was conducted in accordance with the Preferred Reporting Items for Systematic Reviews and Meta-Analyses (PRISMA 2020) statement and EQUATOR Network recommendations [31,32]. The research question, eligibility criteria, search strategy, study-selection process, data items, and planned synthesis were defined a priori before screening began.

The protocol was developed with reference to PRISMA-P guidance [33] but was not prospectively registered in PROSPERO or another public registry. This absence of prospective registration is acknowledged as a methodological limitation.

The protocol specified a qualitative synthesis of the prognostic significance, biomarker landscape, clinical applicability, and therapeutic implications of TCGA-based taxonomy and clinically applicable classifiers, including ProMisE and TransPORTEC.

### 2.2. Search Strategy

A comprehensive systematic literature search was performed in the following electronic databases:

- PubMed/MEDLINE;
- Embase;
- Scopus;
- Web of Science Core Collection;
- Cochrane Library.

The search covered studies published from 1 January 2013, the year of the TCGA molecular classification, through 30 June 2026.

The search strategy combined Medical Subject Headings (MeSHs), Emtree terms, and free-text terms for endometrial cancer, TCGA/ProMisE classification, POLE, MMR protein loss, MSI testing, p53 immunohistochemistry, TP53 sequencing, prognosis, biomarkers, and treatment.

The complete database-specific search syntax, including field tags, Boolean operators, date limits, and language limits, is provided in the Supplementary Materials.

Search strings were adapted to the indexing system and syntax of each database. MMR immunohistochemistry and MSI testing terms were included separately, as were p53 immunohistochemistry and TP53 genomic terms, to avoid treating related assays as exact synonyms.

Reference lists of eligible primary studies and relevant secondary sources were also screened to identify additional records. Clinical guidelines, consensus statements, systematic reviews, and meta-analyses were used for contextual interpretation and citation chasing but were not included in the formal evidence set.

Trial registries and other gray-literature sources were screened to identify potentially eligible full-text reports; unpublished and abstract-only records were not included in the formal evidence synthesis. No geographic restrictions were applied.

Only English-language publications were included. This language restriction was considered when interpreting potential selection bias.

### 2.3. Eligibility Criteria

Eligibility criteria were defined according to the Population, Exposure/Intervention, Comparator, Outcomes, and Study Design (PICOS) framework.

Inclusion Criteria

Studies were eligible for inclusion if they met the following criteria:

1. Included patients with histologically confirmed endometrial carcinoma.
2. Reported original primary research rather than a guideline, review, meta-analysis, commentary, or expert synthesis.
3. Evaluated TCGA, ProMisE, TransPORTEC, or an equivalent four-group molecular classifier.
4. Specified the assay used to determine POLE status, MMR protein expression and/or MSI status, and p53 immunohistochemical and/or TP53 genomic status.
5. Reported at least one prognostic, predictive, diagnostic, biomarker-related, or therapeutic outcome.
6. Used a prospective or retrospective cohort, randomized trial or trial-based molecular analysis, diagnostic/validation, translational, or population-based design and was published in a peer-reviewed journal from 2013 through June 2026.

Exclusion Criteria

Studies were excluded if they:

1. Were case reports or case series with fewer than ten patients.
2. Were conference abstracts without a full-text publication.
3. Were editorials, commentaries, narrative or systematic reviews, meta-analyses, clinical guidelines, consensus statements, letters, or expert opinions; these sources could be used only for clinical context or reference screening.
4. Included only non-human experimental models without clinical patient data.
5. Did not provide sufficient information on molecular classification methods or clinically relevant outcomes.
6. Duplicated or substantially overlapped a previously reported patient cohort without providing distinct outcomes.

### 2.4. Screening and Selection Process

All identified records were collated, and duplicates were removed before screening.

Two reviewers independently screened titles and abstracts. Potentially eligible reports underwent full-text assessment against the predefined criteria.

Disagreements were resolved through discussion and consensus; a third senior reviewer adjudicated unresolved decisions.

**Figure 1:**
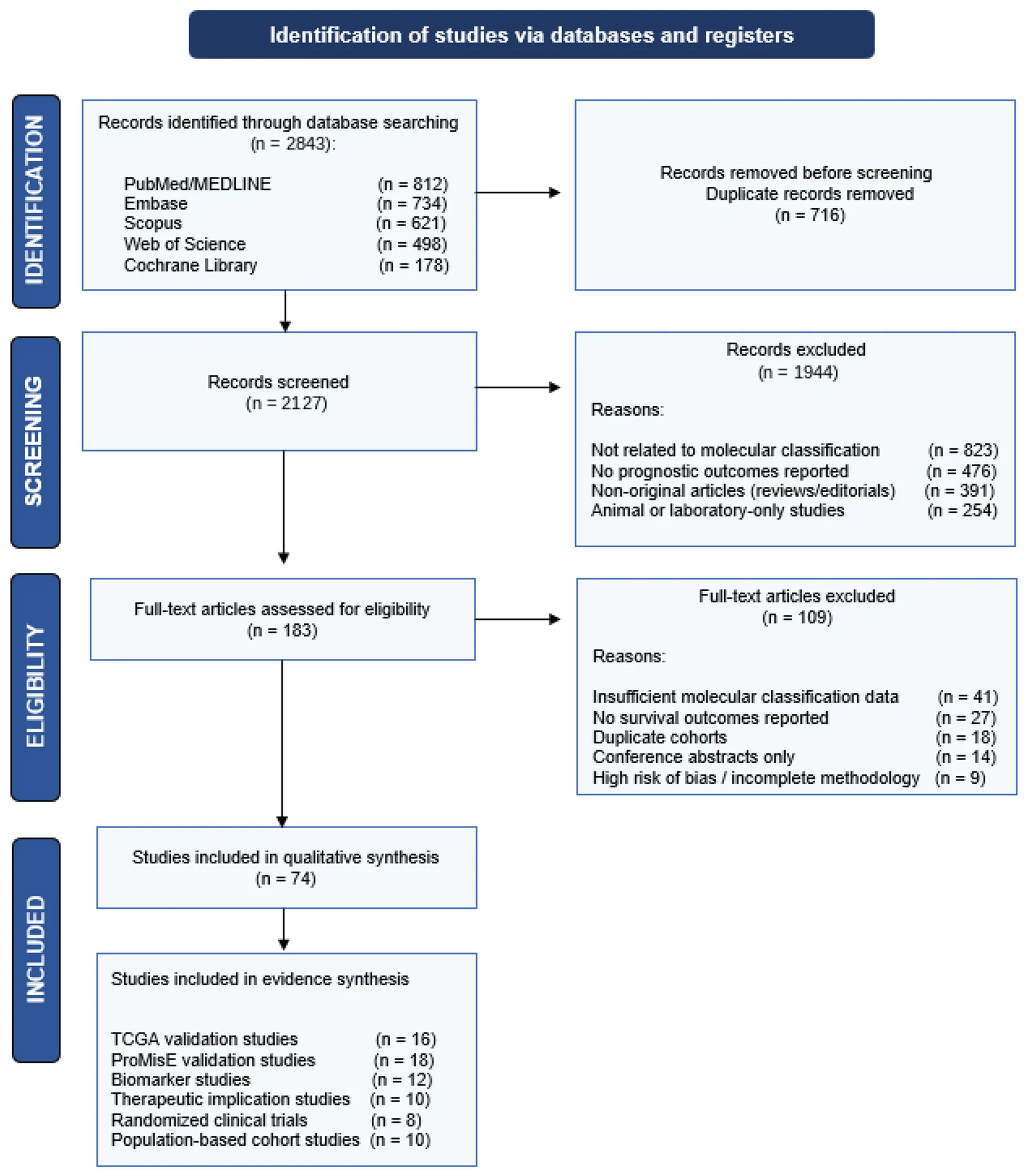
PRISMA 2020 flow diagram showing study identification, screening, full-text eligibility assessment, and inclusion in the qualitative synthesis.

The study selection process was documented using a PRISMA 2020 flow diagram (Figure 1).

### 2.5. Data Extraction

Two investigators independently extracted data using a standardized form developed for this review.

The following variables were collected:

- First author;
- Year of publication;
- Country;
- Study design;
- Number of participants;
- Histological subtype;
- Molecular classifier used;
- Assay used for MMR status (IHC) and/or MSI status (PCR or NGS);
- p53 immunohistochemical pattern and/or TP53 sequencing result;
- POLE variant and reported pathogenicity;
- Presence and handling of multiple-classifier features;
- Molecular subgroup distribution;
- Biomarkers evaluated;
- Follow-up duration;
- Overall survival (OS);
- Disease-specific survival (DSS);
- Progression-free survival (PFS);
- Recurrence-free survival (RFS);
- Hazard ratios (HRs);
- Therapeutic interventions;
- Main conclusions.

Extraction discrepancies were resolved by re-examining the source publication and reaching consensus.

### 2.6. Quality Appraisal and Risk of Bias Tools

Two reviewers independently assessed methodological quality and risk of bias using design-appropriate tools.

Diagnostic and classifier-validation studies were assessed with QUADAS-2 [34], which evaluates patient selection, the index test, the reference standard, and flow and timing.

The QUADAS-2 framework evaluates four domains:

- Patient selection and spectrum;
- Prespecification and interpretation of the index test;
- Appropriateness of the reference standard;
- Completeness of flow and timing.

Each QUADAS-2 domain was rated as low, high, or unclear risk of bias, with applicability concerns considered separately.

Observational cohort studies were evaluated with the Newcastle–Ottawa Scale, and randomized trials were assessed with RoB 2 [35,36]. Translational studies that did not fit these designs were appraised descriptively for cohort selection, assay reporting, reproducibility, prespecification, and external validation.

Disagreements in quality assessment were resolved by consensus.

### 2.7. Data Synthesis

Because of expected and observed heterogeneity in study design, molecular assays, patient populations, treatment exposure, outcome definitions, and follow-up, a narrative synthesis was prespecified as the primary analytical approach.

Evidence was interpreted according to the question addressed by each design: randomized trials primarily informed treatment effects; prospective and retrospective cohorts informed prognostic associations; diagnostic and validation studies informed classifier performance and assay concordance; and translational studies informed biological plausibility. Findings were grouped by molecular subgroup, biomarker, and therapeutic context.

When findings differed, potential explanations were examined, including stage and histologic composition, assay platform and variant classification, biomarker cutoffs, treatment protocols, follow-up duration, and adequacy of multivariable adjustment. A quantitative meta-analysis was not performed because sufficiently homogeneous estimates were not available. No formal GRADE assessment or single overall certainty rating was assigned.

## 3. Results

### 3.1. Study Selection

The systematic search identified 2843 records across the selected databases, including PubMed/MEDLINE (*n* = 812), Embase (*n* = 734), Scopus (*n* = 621), Web of Science (*n* = 498), and the Cochrane Library (*n* = 178). Following removal of 716 duplicate records, 2127 studies underwent title and abstract screening.

After title and abstract screening, 1944 records were excluded because they did not address molecular classification, lacked clinically relevant outcomes, were non-original publications, or involved non-human models. A total of 183 full-text reports were assessed for eligibility.

Of these, 109 reports were excluded because of insufficient molecular-classification data (*n* = 41), absence of relevant prognostic or therapeutic outcomes (*n* = 27), overlapping cohorts (*n* = 18), conference abstracts without full publications (*n* = 14), or major methodological limitations (*n* = 9).

Ultimately, 74 primary studies met the eligibility criteria and were included in the qualitative synthesis. The evidence base comprised classifier-validation and diagnostic studies, prognostic cohorts, translational biomarker studies, randomized trials or trial-based molecular analyses, and population-based cohorts. These design categories addressed different clinical questions and were interpreted accordingly.

### 3.2. Characteristics of Included Studies

The 74 included studies involved approximately 42,000 patients with histologically confirmed endometrial carcinoma and were published from 2013 through June 2026.

Selected primary studies representing the major evidence domains are summarized in Table 1. Clinical guidelines and secondary evidence were reviewed separately for clinical context and were not counted among the 74 included studies.

Most studies originated from North America, Europe, and East Asia. Retrospective cohorts were the most common design, followed by prospective cohorts, translational or classifier-validation studies, randomized trials or trial-based molecular analyses, and population-based investigations.

Molecular classification used TCGA-scale genomic profiling, ProMisE or TransPORTEC algorithms, targeted NGS, and immunohistochemistry-based surrogates. MMRd was identified principally by loss of MLH1, PMS2, MSH2, and/or MSH6 on IHC; some studies also or instead reported MSI-H by PCR or NGS. p53abn was usually based on abnormal p53 IHC, whereas a subset of studies used TP53 sequencing. These assay results were extracted and interpreted separately.

Across studies, reported subgroup distributions were broadly consistent with established population ranges, although estimates varied with histologic composition, stage, and testing algorithm:

- POLE-mutated: approximately 7–12%;
- MMR-deficient: approximately 25–35%;
- No specific molecular profile: approximately 40–50%;
- p53-abnormal: approximately 15–25%.

The principal outcomes assessed included overall survival (OS), progression-free survival (PFS), disease-specific survival (DSS), recurrence-free survival (RFS), response to systemic therapy, and predictive value of molecular biomarkers.

### 3.3. Prognostic Significance of Molecular Subgroups

#### 3.3.1. POLE-Ultramutated Endometrial Carcinoma

In most validation cohorts, tumors with pathogenic POLE exonuclease-domain mutations had the most favorable outcomes among the four molecular groups [14,15,16,17,21,39].

TransPORTEC and PORTEC-3 analyses reported very low recurrence and disease-specific mortality among POLEmut tumors, including many high-grade cases; however, estimates were based on relatively small subgroup numbers, and evidence in advanced-stage disease remains limited [15,39].

The favorable prognosis is biologically consistent with an ultramutated phenotype, high neoantigen burden, and prominent antitumor immune responses [40]. In multiple-classifier tumors, a pathogenic POLE mutation generally appears to dominate the adverse signal of concurrent p53 abnormality [21].

These findings support investigation of treatment de-escalation, but omission or reduction in adjuvant therapy should follow validated risk algorithms, guideline-supported pathways, or clinical-trial protocols rather than POLE status in isolation [15,21,39].

#### 3.3.2. Mismatch Repair-Deficient Endometrial Carcinoma

MMRd tumors represented approximately one-quarter to one-third of cases in most cohorts, although prevalence varied by age, histology, and the testing algorithm used.

Prognostic results for MMRd were less uniform than for POLEmut or p53abn tumors. Outcomes were generally intermediate, but differences across studies likely reflected stage, histologic composition, sporadic MLH1 promoter methylation versus Lynch-associated disease, treatment exposure, and follow-up duration.

MMRd and MSI-H describe related biologic phenomena but different analytical results. Loss of one or more MMR proteins is identified by IHC, whereas MSI-H is detected by PCR- or NGS-based assays; concordance is usually high, but discordant cases occur and require method-specific reporting and review [20].

The predictive evidence for immune checkpoint inhibition was stronger and more consistent than the purely prognostic evidence. GARNET, RUBY, NRG-GY018, and AtTEnd demonstrated clinically meaningful activity or treatment benefit in appropriate advanced or recurrent disease settings defined by MMRd or MSI-H [25,26,37,38].

#### 3.3.3. No Specific Molecular Profile (NSMP)

NSMP was the largest and most heterogeneous molecular category. Reported outcomes ranged from favorable to high risk, indicating that the absence of POLEmut, MMRd, and p53abn does not define a biologically uniform disease [28,29,41].

Within NSMP, CTNNB1 exon 3 mutations have been associated with increased recurrence risk, whereas strong ER and PR expression generally identifies a more favorable, hormone-responsive phenotype. L1CAM expression is associated with adverse features and poorer outcomes [29,42,43,44,45,46].

ARID1A loss and chromosome 1q alterations may provide additional context, but their incremental value is not yet standardized. Taken together, ER/PR, CTNNB1, L1CAM, ARID1A, and chromosome 1q status may support a multivariable refinement model rather than acting as interchangeable single markers [28,29,41,47,48,49,50].

Most NSMP-refinement evidence is retrospective, uses heterogeneous assays and cutoffs, and lacks prospective treatment interaction data. These biomarkers should therefore be considered investigational for formal risk-group assignment.

#### 3.3.4. p53-Abnormal Endometrial Carcinoma

p53abn tumors were generally associated with the least favorable prognosis. In clinically applicable classifiers, p53abn is defined by an abnormal p53 immunohistochemical pattern, including diffuse overexpression, complete absence in tumor cells with an intact internal control, or an abnormal cytoplasmic pattern [18,19,51].

Abnormal p53 IHC is strongly associated with pathogenic TP53 alterations, but the two terms are not exact synonyms. Rare sequencing-IHC discordance, technical artifacts, and subclonal abnormal staining can occur, particularly in multiple-classifier tumors [18,19,51].

Across cohorts, p53abn status was associated with chromosomal instability, frequent HER2 amplification, higher recurrence risk, and inferior survival. In the PORTEC-3 molecular analysis, the p53abn subgroup appeared to derive the greatest benefit from combined chemoradiotherapy, although treatment decisions remain dependent on stage and other clinicopathologic factors [15,51].

Accordingly, reports should specify whether p53 abnormality was defined by IHC or whether a TP53 alteration was sequencing-confirmed, and discordant or subclonal results should be reviewed before assigning the molecular group.

#### 3.3.5. Multiple-Classifier Endometrial Carcinomas

A small proportion of endometrial carcinomas harbor more than one molecular classifying feature, most commonly POLEmut-p53abn, MMRd-p53abn, POLEmut-MMRd, or, rarely, all three. In the cohort reported by León-Castillo et al., 107 of 3518 profiled tumors (3%) showed p53 abnormality together with another classifier. POLEmut-p53abn tumors clustered molecularly and clinically with POLEmut tumors, whereas MMRd-p53abn tumors more closely resembled MMRd tumors [21].

These findings support a practical hierarchical approach: assign a pathogenic POLE exonuclease-domain mutation first; if POLE is wild type, assign MMR deficiency next; assign p53abn only when POLE is wild type and MMR is proficient; and define NSMP by exclusion. Thus, a tumor with a pathogenic POLE mutation and an abnormal p53 immunohistochemical pattern is generally classified as POLEmut rather than p53abn [21,39].

Before hierarchical assignment, the pathogenicity of the POLE variant, staining quality, internal controls, and the possibility of subclonal p53 abnormality should be reviewed. Evidence is derived mainly from retrospective molecular cohorts; treatment selection should therefore remain integrated with stage, histology, clinicopathologic risk factors, and multidisciplinary review.

The defining assays, prognostic patterns, clinical implications, and hierarchy for multiple-classifier tumors are summarized in Table 2.

### 3.4. Risk of Bias Assessment

QUADAS-2 assessments indicated predominantly low-to-moderate risk of bias in diagnostic and classifier-validation studies. The most frequent concerns involved non-consecutive patient selection, incomplete reporting of assay thresholds or failed tests, and variation in the reference standard.

Observational prognostic studies were generally of moderate-to-high quality on the Newcastle–Ottawa Scale but remained vulnerable to retrospective selection, treatment confounding, variable follow-up, and incomplete multivariable adjustment.

**Figure 2:**
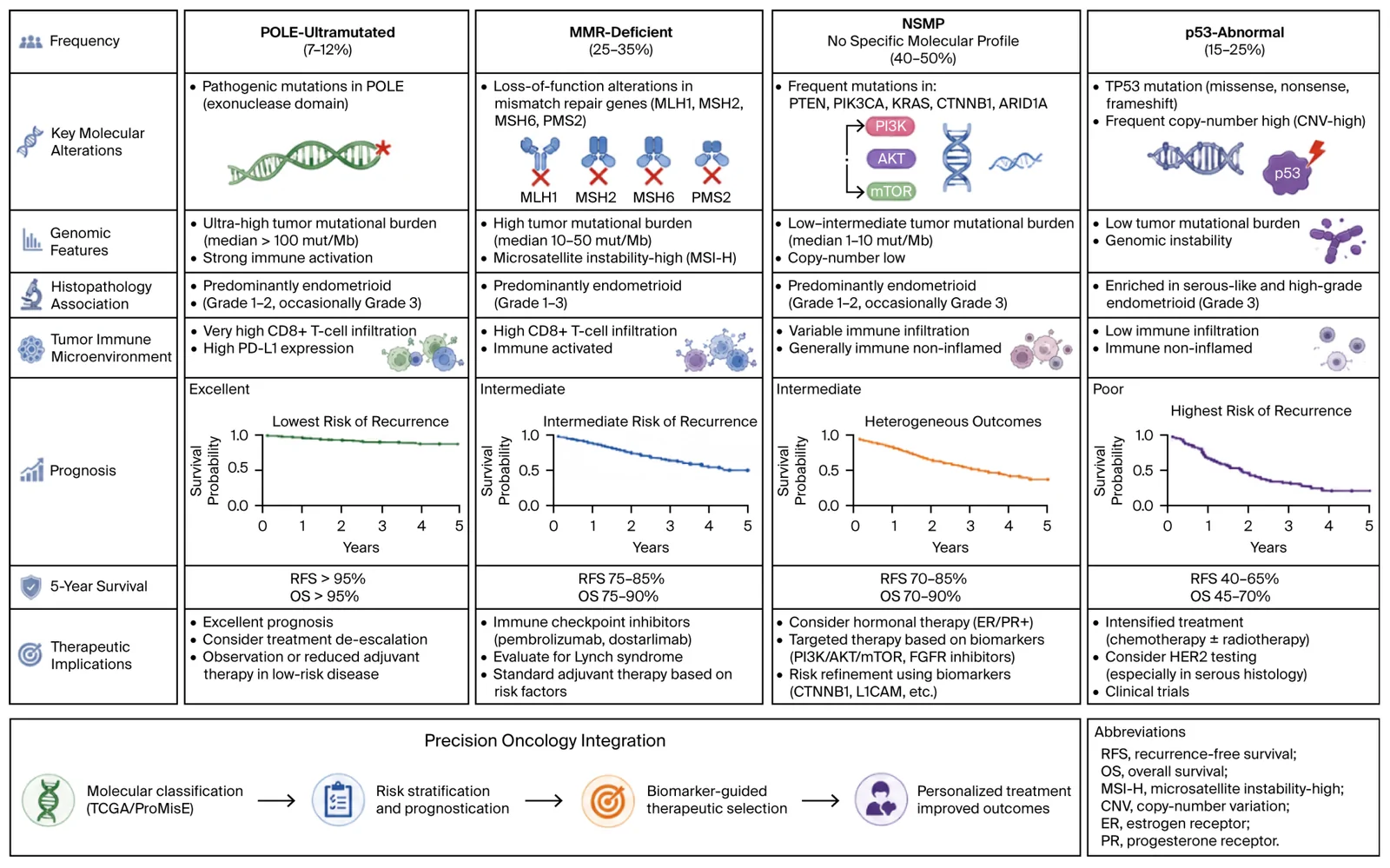
Molecular landscape of endometrial cancer. In this review, MMRd denotes loss of MMR protein expression by immunohistochemistry, whereas MSI-H denotes a related but analytically distinct PCR- or NGS-based result. p53abn denotes an abnormal immunohistochemical pattern; TP53 mutation is closely associated but not synonymous.

Parent randomized trials generally had low risk of bias under RoB 2; however, molecular subgroup analyses were sometimes limited by subgroup size, multiplicity, and post hoc interaction testing. Translational studies mainly provided mechanistic support and were not treated as equivalent to prospective clinical-effect evidence. The molecular landscape of the four principal molecular groups is summarized in Figure 2, and a design-level summary of the risk-of-bias assessment is provided in Table 3.

Because no formal GRADE assessment was performed and the evidence base was heterogeneous, no single overall certainty rating was assigned. Conclusions were weighted qualitatively according to study design, risk of bias, consistency, directness, and clinical applicability.

### 3.5. Complementary Biomarkers: Clinical Maturity and Applicability

Additional biomarkers differ substantially in clinical maturity. POLE, MMR/MSI, and p53 testing are core components of molecular classification; HER2 testing is clinically actionable in selected advanced or recurrent serous/p53abn tumors. ER/PR assessment can inform hormonal-treatment selection, whereas CTNNB1, L1CAM, chromosome 1q, FGFR2, PI3K-pathway alterations, and ARID1A remain investigational for formal molecular risk assignment [22,23,24,25,26,27,28,29,30,41,42,43,44,45,46,47,48,49,50,51,52,53,54,55,56,57,58,59,60,61,62,63,64,65].

The analytical method, evidence-supported role, and current clinical status of established and emerging biomarkers are summarized in Table 4.

#### 3.5.1. HER2 Amplification and Overexpression

HER2 (ERBB2) amplification or overexpression is enriched in uterine serous and p53abn carcinomas, with prevalence varying substantially by histotype, assay, and positivity threshold [52,53].

HER2-positive tumors frequently show copy-number-high biology and aggressive clinicopathologic features. The randomized phase II trial by Fader et al. showed improved outcomes when trastuzumab was added to carboplatin-paclitaxel in advanced or recurrent HER2-positive uterine serous carcinoma [27].

Trastuzumab deruxtecan has also shown activity across HER2-expressing solid tumors, including endometrial cancer cohorts [54]. HER2 is therefore clinically actionable in selected settings, but testing method, histologic context, and treatment indication should be specified rather than generalized to all EC.

#### 3.5.2. CTNNB1 Mutations

CTNNB1 exon 3 mutations activate Wnt/β-catenin signaling and occur particularly in low-grade endometrioid tumors within the NSMP group [42].

Several cohorts have associated CTNNB1 mutation with increased recurrence despite otherwise favorable clinicopathologic features [43,44].

A systematic review and meta-analysis supported an adverse association with recurrence in early-stage endometrioid EC [45]. Nevertheless, assay methods, cohort composition, and treatment-adjusted effect estimates vary, and CTNNB1 is not yet a universally standardized clinical risk marker.

#### 3.5.3. PTEN Alterations

PTEN alterations are common in endometrioid EC and may arise early in tumorigenesis [56,57].

PTEN loss activates PI3K/AKT/mTOR signaling, but PTEN alone has shown limited and inconsistent prognostic discrimination [58].

mTOR-directed and combination strategies have shown variable activity, so PTEN status should currently be viewed mainly as a pathway marker and clinical-trial variable rather than a standalone treatment-selection test [59].

#### 3.5.4. PIK3CA Mutations

PIK3CA mutations are frequent in EC and often coexist with PTEN loss, reflecting convergent activation of the PI3K/AKT pathway [60,61].

Associations between PIK3CA alterations and prognosis have differed across histologic and molecular contexts, limiting their use as independent risk markers.

PI3K/AKT/mTOR inhibitors remain under investigation in molecularly selected populations. Outside a validated indication or clinical trial, PIK3CA status should not be assumed to predict benefit [62].

#### 3.5.5. FGFR2 Alterations

FGFR2 mutations occur in a minority of endometrioid ECs and may be enriched in MSI/MMRd-associated tumors [63,64].

FGFR2 activation affects proliferative, angiogenic, and survival pathways. Prognostic associations have varied across cohorts and require molecular-context adjustment [64].

Early-phase studies of FGFR inhibitors have shown activity in selected FGFR-altered solid tumors [65]. In EC, FGFR2 remains a clinical-trial biomarker rather than an established routine treatment-selection marker.

#### 3.5.6. ARID1A Inactivation

ARID1A is a component of the SWI/SNF chromatin-remodeling complex. Loss-of-function alterations are common in endometrioid EC and frequently occur in MMRd or MSI-H tumors [47].

Preclinical and translational studies link ARID1A loss to genomic instability, altered DNA-damage responses, and potential synthetic-lethal or immunologic vulnerabilities [48,49].

Clinical predictive value has not been prospectively established. ARID1A should therefore remain an investigational biomarker rather than a basis for routine therapy selection.

#### 3.5.7. Other Investigational Biomarkers

Additional candidates discussed in contemporary secondary literature include CCNE1 amplification, homologous-recombination deficiency, NTRK fusions, POLD1 variants, and epigenetic regulators [30,55]. These sources were used for context and were not counted among the 74 primary studies.

Integrative genomic, transcriptomic, proteomic, and radiomic approaches may identify biologically refined subgroups beyond the four-class framework [66,67]. However, analytical pipelines, sample processing, model calibration, and external validation remain heterogeneous.

Accordingly, biomarkers beyond the core classifier should be presented according to their level of clinical maturity. Evidence of association or druggability does not by itself establish clinical utility, and most emerging markers require prospective validation in molecularly stratified cohorts.

## 4. Discussion

This review synthesizes evidence from 74 primary studies addressing different clinical questions. The findings support the clinical relevance of molecular classification, but they do not justify treating all designs, assays, populations, or endpoints as equally informative. Molecular results should be interpreted as complementary to, rather than a replacement for, stage, histotype, grade, LVSI, and patient factors. Earlier staging, consensus, clinicopathologic, and histopathologic studies also document the evolution toward integrated risk assessment [68,69,70,71,72].

### 4.1. Interpretation of Heterogeneous Evidence

Randomized trials and trial-based molecular analyses provided the most direct evidence for treatment effects, whereas observational cohorts primarily informed prognosis, diagnostic studies informed assay concordance, and translational studies supported biological plausibility. This design-based separation was essential because a prognostic association does not necessarily predict treatment benefit.

Several apparently contradictory findings could be explained by differences in stage, histologic composition, assay platform, POLE variant classification, p53 or MMR interpretation, treatment exposure, endpoint definition, and follow-up. MMRd prognosis, for example, varied more across cohorts than its predictive association with immunotherapy, while NSMP results depended strongly on hormone-receptor and additional biomarker composition.

**Figure 3:**
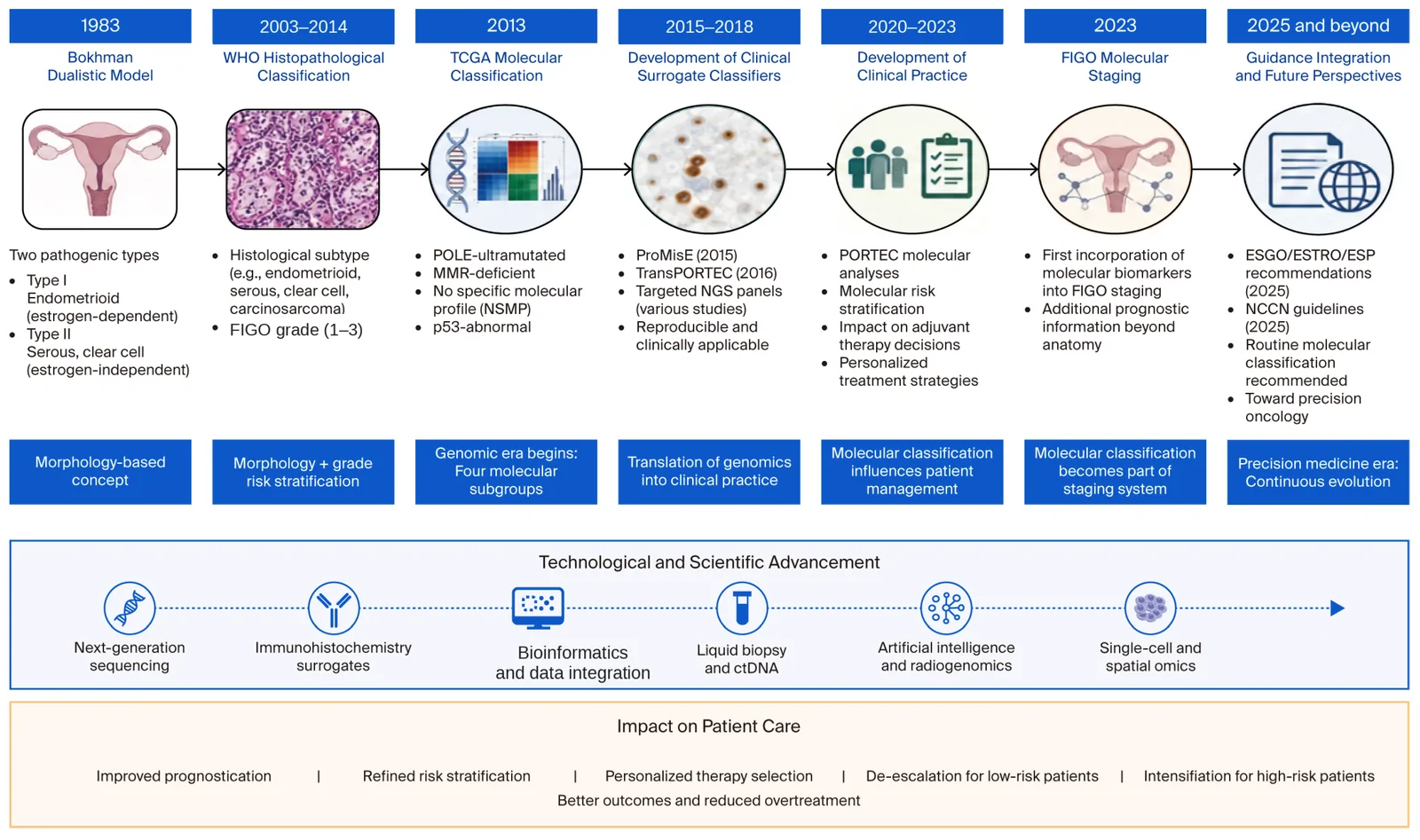
Evolution from histopathological to molecular classification of endometrial cancer.

The most reproducible contrasts were the favorable outcome of pathogenic POLEmut tumors and the adverse outcome of many p53abn tumors. Even these conclusions require caution because POLEmut subgroups are small, advanced-stage data are limited, and p53 IHC may be subclonal or discordant with sequencing. The evolution of classification from morphology to integrated molecular-pathologic assessment is illustrated in Figure 3.

### 4.2. Clinical Significance and Remaining Uncertainty

Pathogenic POLEmut tumors generally have favorable outcomes, supporting careful study of de-escalation. However, variant pathogenicity must be established, and de-escalation should not be extrapolated indiscriminately to nonpathogenic POLE variants, advanced-stage disease, or unvalidated clinical settings [15,21,39,40].

For MMRd tumors, prognostic estimates were heterogeneous, whereas predictive evidence for immune checkpoint inhibition was comparatively robust in advanced and recurrent disease [25,26,37,38]. MMR IHC and MSI testing should be reported separately, and discordant results require review rather than automatic relabeling [20].

NSMP should not be interpreted as a single intermediate-risk entity. ER/PR expression, CTNNB1, L1CAM, chromosome 1q, and ARID1A may collectively define more favorable and less favorable subsets, but no universally validated composite algorithm currently exists [28,29,41,42,43,44,45,46,47,48,49,50].

For p53abn tumors, abnormal IHC is an established surrogate classifier, not a synonym for a sequencing-confirmed TP53 alteration. Accurate recognition of overexpression, null, cytoplasmic, and subclonal patterns is essential because misclassification may alter risk assignment [18,19,51].

Overall, the evidence supports integrated molecular-pathologic risk assessment. It is strongest when a biomarker is analytically defined, prognostically replicated, and linked to treatment benefit in an appropriate trial population; it is weaker when based only on retrospective association or preclinical actionability.

### 4.3. Multiple-Classifier Tumors and Molecular Hierarchy

Multiple-classifier tumors illustrate why molecular labels cannot always be assigned independently. The largest foundational cohort showed that POLEmut-p53abn tumors behaved more like POLEmut tumors and MMRd-p53abn tumors more like MMRd tumors [21].

A practical hierarchy therefore assigns pathogenic POLEmut first, MMRd second, and p53abn third; NSMP is assigned only when the other features are absent. For example, a tumor with a pathogenic POLE mutation and abnormal p53 IHC is generally classified as POLEmut, after confirming variant pathogenicity and excluding technical or interpretive error [21,39].

This hierarchy improves prognostic coherence but does not eliminate uncertainty. Subclonal p53 abnormality, equivocal staining, discordant MMR/MSI results, or a POLE variant of uncertain significance should trigger pathology and molecular review rather than automatic classification.

Prospective treatment-interaction data for multiple-classifier tumors remain limited. Treatment decisions should therefore incorporate stage, histology, other risk factors, and multidisciplinary judgment rather than relying on hierarchy alone.

### 4.4. Clinical Guidelines and Current Applicability

The ESGO/ESTRO/ESP and NCCN guidance cited in this review and the FIGO 2023 staging framework incorporate molecular information into risk assessment and treatment planning [22,23,24]. These documents were used for clinical context and were not included in the formal set of 74 primary studies.

Guideline integration does not imply that every biomarker discussed in this review is ready for routine use. POLE sequencing, MMR IHC, MSI testing, or both, and p53 IHC have the clearest role in molecular assignment; HER2 is actionable in selected advanced or recurrent serous/p53abn settings [22,23,24,25,26,27].

Potential de-escalation for POLEmut disease and treatment intensification for p53abn disease should follow the specific stage, risk group, and evidence context. Likewise, immunotherapy eligibility depends on disease setting, treatment line, regulatory context, and the exact MMR/MSI assay rather than a generic molecular label.

ER/PR, CTNNB1, L1CAM, chromosome 1q, ARID1A, FGFR2, and PI3K-pathway alterations may refine biological understanding or trial eligibility, but most are not yet validated for routine reassignment of adjuvant risk groups.

**Figure 4:**
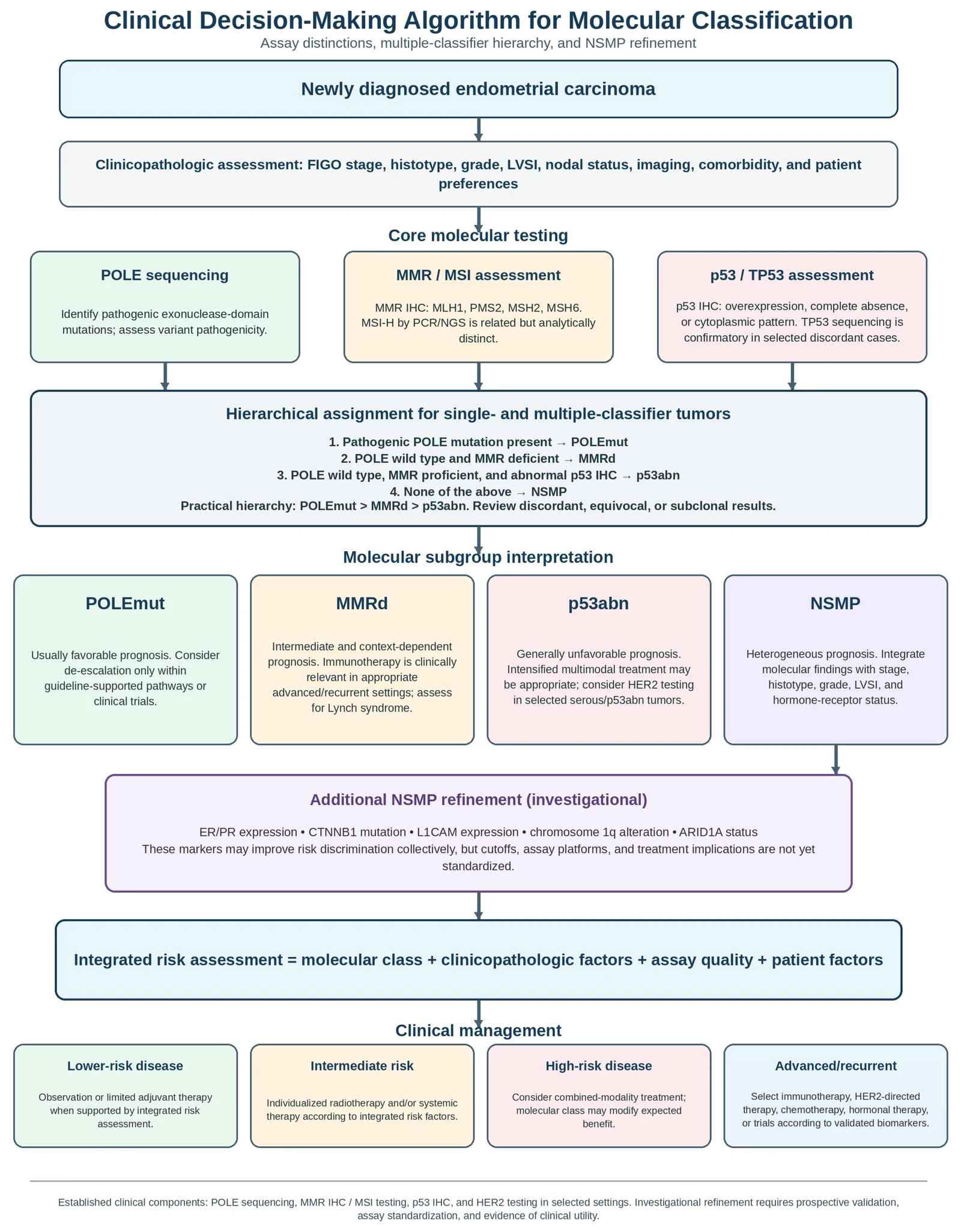
Clinical decision-making algorithm incorporating assay distinctions, the hierarchy for multiple-classifier tumors, and investigational NSMP refinement. Treatment decisions must remain integrated with clinicopathologic factors and the applicable guideline or regulatory context.

Figure 4 presents a practical algorithm that separates the relevant assays, incorporates the hierarchy for multiple-classifier tumors, and distinguishes established clinical components from investigational NSMP refinement.

Clinical decisions should remain integrated with morphology, stage, LVSI, nodal status, comorbidity, treatment goals, and patient preferences.

### 4.5. Implementation Challenges and Emerging Technologies

Routine implementation is constrained by tissue quality, turnaround time, cost, reimbursement, laboratory capacity, and access to gynecologic pathology and molecular expertise. Standardized preanalytics, validated assays, internal controls, external quality assurance, and structured reporting are prerequisites for reliable classification.

Assay discordance is a practical challenge. MMR IHC and MSI testing may disagree, p53 IHC may be equivocal or subclonal, and nonpathogenic POLE variants may be misinterpreted. Multidisciplinary review and confirmatory testing should be available for clinically consequential discordant results [18,19,20,39].

Liquid biopsy, artificial intelligence, radiogenomics, and multi-omics approaches are promising but remain largely investigational. Before clinical use, they require multicenter external validation, locked algorithms and cutoffs, calibration across populations, demonstration of incremental value over standard care, and prospective evidence that their use improves decisions or outcomes [66,67,73,74,75,76,77,78].

Digital and multi-omics implementation also raises issues of data interoperability, reproducibility, explainability, regulatory oversight, privacy, and equitable access. Performance in a development cohort should not be equated with transportable clinical utility.

A feasible implementation pathway is a tiered workflow: validated core classification for all appropriate cases, reflex confirmatory testing for discordant results, and additional biomarkers only when linked to a defined clinical question, guideline indication, or clinical trial.

### 4.6. Strengths and Limitations

Strengths include adherence to PRISMA 2020 reporting, searches across five major databases, explicit separation of primary evidence from guidelines and secondary syntheses, inclusion of prognostic and therapeutic studies, and use of design-appropriate risk-of-bias tools.

Limitations include the absence of prospective protocol registration, restriction to English-language publications, substantial heterogeneity in assays, populations, treatment protocols, outcome definitions, and follow-up, and the inability to perform a meaningful quantitative meta-analysis.

No formal GRADE assessment was performed; therefore, this review does not assign a single high-certainty rating to the evidence. Some biomarker findings were derived from retrospective, selected, or underpowered subgroups, and recently reported trials have limited long-term follow-up.

The literature is also rapidly evolving, and technologies or indications may change after the search cutoff. The conclusions should therefore be interpreted as a structured qualitative synthesis, with the strongest weight given to replicated, methodologically sound, and clinically direct evidence.

## 5. Research Priorities and Future Perspectives

Future research should move beyond biomarker association toward analytically validated, prospectively tested, and clinically useful tools. Priority areas include NSMP refinement, multi-omics integration, minimal-residual-disease monitoring, digital pathology, radiogenomics, spatial technologies, and molecularly adaptive trials.

### 5.1. Refinement of the NSMP Molecular Subgroup

NSMP remains the least biologically defined group. Integrated models combining ER/PR, CTNNB1, L1CAM, chromosome 1q, ARID1A, and clinicopathologic variables may improve discrimination [28,29,41,46,50].

The next step is prospective, multicenter validation using prespecified assays, cutoffs, and endpoints, followed by treatment-interaction studies. A marker should not be incorporated into a routine algorithm until it shows reproducibility, incremental value, and a clinically actionable consequence.

### 5.2. Integration of Multi-Omics Approaches

Multi-omics can reveal mechanisms of progression and resistance by integrating genomic, transcriptomic, epigenomic, proteomic, metabolomic, and spatial data [67].

Major barriers include tissue and processing variability, batch effects, high dimensionality, missing data, overfitting, computational cost, and limited external validation. Shared data standards, transparent pipelines, independent validation, and prospective utility studies are required before routine use.

### 5.3. Liquid Biopsy and Minimal Residual Disease Monitoring

ctDNA and other liquid-biopsy analytes may support postoperative risk assessment, treatment monitoring, and detection of molecular recurrence [73,74,77].

Current studies are small and use heterogeneous platforms, sampling schedules, and positivity thresholds. Sensitivity may be limited in low-volume early-stage disease, and earlier molecular detection has not yet been proven to improve survival.

Prospective trials should standardize preanalytics and assay thresholds and test whether ctDNA-guided intervention improves patient-important outcomes rather than only advancing the date of recurrence detection.

### 5.4. Artificial Intelligence and Digital Pathology

Artificial intelligence may predict molecular subtype from digital pathology and integrate pathology, imaging, genomics, and clinical variables [75].

Priority requirements are multicenter external validation, assessment of dataset shift and subgroup performance, explainability appropriate to the clinical task, locked algorithms, workflow integration, and regulatory oversight.

AI should be evaluated against current standard testing and used as an assistive tool until prospective evidence demonstrates safe and reproducible clinical utility.

### 5.5. Radiomics and Radiogenomics

Radiomics and radiogenomics may provide noninvasive estimates of molecular phenotype and whole-tumor heterogeneity [66].

However, image acquisition, reconstruction, segmentation, feature extraction, and model calibration vary across centers, creating substantial reproducibility risk.

Future studies should use standardized imaging protocols, transparent feature definitions, external validation, and prospective comparison with tissue-based testing before clinical deployment.

### 5.6. Single-Cell and Spatial Transcriptomic Technologies

Single-cell and spatial transcriptomic technologies can characterize intratumoral heterogeneity, clonal evolution, and tumor-microenvironment interactions [78].

Translation is limited by tissue-handling sensitivity, cost, computational complexity, sampling bias, and lack of standardized clinical interpretation. Near-term value is likely to lie in target discovery and resistance biology rather than routine classification.

### 5.7. Molecularly Adaptive Clinical Trials

Biomarker-driven umbrella, basket, and adaptive platform trials can test multiple molecularly selected strategies efficiently [79].

Design challenges include rare subgroups, assay harmonization, changing standards of care, multiplicity, access to testing, and interpretation of treatment interactions. Prospective collection of tissue and blood and prespecified molecular analyses should be embedded in trials.

Collectively, these technologies may refine precision oncology, but their adoption should be contingent on analytical validity, reproducibility, external validation, feasibility, clinical utility, and equitable implementation.

## 6. Conclusions

Molecular classification has substantially changed the biologic interpretation and clinical risk assessment of endometrial cancer. TCGA-derived and clinically applicable classifiers distinguish groups with different prognostic patterns and, in selected settings, different treatment implications.

Across the 74 included primary studies, pathogenic POLEmut tumors generally had favorable outcomes, p53abn tumors generally had unfavorable outcomes, and MMRd and NSMP tumors showed more context-dependent heterogeneity. Molecular classification usually added information beyond clinicopathologic assessment, but the magnitude and directness of evidence varied by design and clinical question.

Contemporary ESGO/ESTRO/ESP and NCCN guidance and the FIGO 2023 framework incorporate molecular information [22,23,24]. Clinical application should remain integrated with stage, histology, grade, LVSI, nodal status, treatment setting, and patient factors rather than being interpreted as a standalone mandate.

POLE sequencing, MMR IHC, MSI testing, or both, and p53 IHC have the clearest roles in molecular assignment; HER2 is actionable in selected tumors. MMRd and MSI-H—and p53abn and TP53 mutation—are closely related but analytically distinct concepts that should be reported precisely.

Multiple-classifier tumors should generally be interpreted using the hierarchy POLEmut > MMRd > p53abn after confirming assay quality and variant pathogenicity. NSMP refinement using ER/PR, CTNNB1, L1CAM, chromosome 1q, ARID1A, and other biomarkers is promising but remains insufficiently standardized for universal routine use.

Future progress will depend not only on technological innovation but also on prospective validation, assay standardization, reproducible reporting, demonstration of clinical utility, and equitable implementation.

In conclusion, molecular classification is an important component of contemporary EC management, but the evidence supports calibrated rather than absolute claims. The greatest clinical value is achieved when validated molecular results are combined with clinicopathologic factors and interpreted within the relevant treatment and guideline context.

## Figures and Tables

**Table 1:** Selected Primary Studies from the Formal Evidence Synthesis.

Study	Year	Design	Population (n)	Classifier/Assay	Contribution to the Evidence Synthesis
TCGA Research Network [14]	2013	Integrated genomic study	373	TCGA	Defined four genomic molecular groups.
Talhouk et al. [16]	2015	Classifier-development study	319	ProMisE	Developed a clinically applicable surrogate classifier.
León-Castillo et al., PORTEC-3 [15]	2020	Trial-based molecular analysis	410	TCGA surrogate	Evaluated subgroup-specific prognosis and treatment effects.
Kommoss et al. [17]	2018	Population-based validation	947	ProMisE	Confirmed reproducibility and prognostic separation.
Singh et al. [18]	2020	Diagnostic concordance study	207	p53 IHC vs. TP53 NGS	Assessed p53 IHC as a surrogate for TP53 mutation.
Smithgall et al. [20]	2022	Discordance study	328	MMR IHC, MSI-PCR, NGS	Investigated analytically discordant MMR/MSI results.
RUBY trial [26]	2023	Phase III randomized trial	494	MMR/MSI subgroup	Evaluated dostarlimab plus chemotherapy.
Fader et al. [27]	2020	Randomized phase II trial	61	HER2-positive serous EC	Evaluated trastuzumab added to carboplatin-paclitaxel.
Vermij et al. [29]	2023	Prognostic cohort	648	NSMP + ER IHC	Refined prognosis within high-risk NSMP tumors.
NRG-GY018 [37]	2023	Phase III randomized trial	816	MMR subgroup	Evaluated pembrolizumab plus chemotherapy.
AtTEnd [38]	2024	Phase III randomized trial	551	MMR subgroup	Evaluated atezolizumab plus chemotherapy.

Abbreviations: EC, endometrial cancer; IHC, immunohistochemistry; MMR, mismatch repair; NGS, next-generation sequencing; TCGA, The Cancer Genome Atlas. Note: The table lists selected primary studies only. Guidelines and secondary syntheses were used for clinical context and were not part of the formal evidence set.

**Table 2:** Defining Assays, Prognostic Patterns, and Clinical Interpretation of the Four Molecular Groups.

Characteristic	POLEmut	MMRd	NSMP	p53abn
Approximate frequency	7–12%	25–35%	40–50%	15–25%
Defining assay	Pathogenic POLE exonuclease-domain mutation by sequencing	Loss of one or more MMR proteins by IHC; MSI-H may support but is not identical	No pathogenic POLE mutation, MMR proficient, and p53 wild-type pattern	Abnormal p53 IHC: overexpression, complete absence, or cytoplasmic pattern; TP53 mutation is related but not identical
Typical biology	Ultramutated; very high tumor mutational burden; immune-rich	Hypermutated; often immune-rich; heterogeneous etiology	Heterogeneous; hormone-receptor and Wnt/β-catenin differences	Copy-number-high/chromosomally unstable; frequent TP53 and HER2 alterations
General prognostic pattern	Usually favorable	Intermediate and context dependent	Heterogeneous	Usually unfavorable
Current clinical relevance	Risk refinement; de-escalation under study	Lynch screening and immunotherapy selection in appropriate settings	Clinicopathologic integration; additional markers remain largely investigational	High-risk refinement; potential benefit from intensified treatment; HER2 assessment in selected tumors
Multiple-classifier rule	Takes precedence when the POLE variant is pathogenic	Takes precedence over p53abn when POLE is wild type	Assigned only after excluding the other three groups	Assigned when POLE is wild type, and MMR is proficient
Representative evidence	[14,15,16,17,21,39,40]	[14,15,16,17,20,25,26,37,38]	[14,16,17,28,29,41,46,50]	[14,15,18,19,27,51,52,53,54]

Abbreviations: IHC, immunohistochemistry; MMR, mismatch repair; MMRd, mismatch repair-deficient; MSI-H, microsatellite instability-high; NSMP, no specific molecular profile; POLEmut, pathogenic POLE-mutated; p53abn, p53-abnormal by immunohistochemistry. Note: Molecular assignment should be integrated with clinicopathologic factors.

**Table 3:** Concise Summary of Risk-of-Bias Assessment by Study Design.

Study Design	Tool	Overall Pattern	Frequent Concerns	Interpretive Role in This Review
Diagnostic/classifier validation	QUADAS-2	Predominantly low-to-moderate risk	Patient selection; incomplete assay reporting; variable reference standards	Informed classifier performance and assay concordance; poorly described methods were interpreted cautiously.
Observational prognosis/biomarker	Newcastle–Ottawa Scale	Moderate-to-high quality overall	Retrospective selection; treatment confounding; variable follow-up; incomplete adjustment	Informed associations and risk estimates, but not causal treatment effects.
Randomized trials/trial-based molecular analyses	RoB 2	Generally low risk for parent trials	Subgroup power; multiplicity; post hoc interaction analyses	Provided the strongest treatment-effect evidence; subgroup findings were interpreted cautiously.
Translational studies	Structured narrative appraisal	Supportive evidence	Selected cohorts; assay reproducibility; lack of external validation	Supported biological plausibility but did not independently justify clinical recommendations.

Abbreviations: QUADAS-2, Quality Assessment of Diagnostic Accuracy Studies-2; RoB 2, revised Cochrane Risk of Bias tool. This design-level summary should be interpreted together with the narrative description of recurrent study-level limitations.

**Table 4:** Established and Investigational Biomarkers Complementing TCGA-Based Classification.

Biomarker/Assay	Molecular Context	Evidence-Supported Role	Current Clinical Status	Representative Evidence
POLE sequencing	POLEmut	Identifies pathogenic exonuclease-domain variants associated with favorable prognosis	Established classifier; variant pathogenicity must be confirmed	[14,15,16,17,21,39,40]
MMR protein IHC	MMRd	Detects loss of MLH1, PMS2, MSH2, and/or MSH6; supports Lynch-syndrome evaluation and treatment selection	Established classifier and clinically used biomarker	[14,15,16,17,20,25,26,37,38]
MSI testing (PCR/NGS)	Often overlaps with MMRd	Detects MSI-H and predicts immunotherapy sensitivity; not identical to MMR IHC	Clinically established in appropriate settings; report assay explicitly	[20,25,26,37,38]
p53 IHC	p53abn	Abnormal overexpression, complete absence, or cytoplasmic pattern serves as a surrogate for TP53 alteration	Established surrogate classifier; review equivocal/subclonal patterns	[18,19,51]
TP53 sequencing	Often p53abn	Confirms pathogenic genomic alteration in selected or discordant cases	Confirmatory/selected use; not synonymous with p53 IHC	[18,51]
HER2 IHC/ISH	Serous/p53abn-enriched	Selects patients for HER2-directed therapy in defined advanced/recurrent settings	Clinically actionable in selected tumors	[27,52,53,54]
ER/PR IHC	NSMP-enriched	Supports hormonal-treatment selection; may refine NSMP prognosis	Clinically used for receptor status; NSMP risk refinement not universally standardized	[28,29,50]
CTNNB1	NSMP	Associated with recurrence risk in otherwise favorable endometrioid tumors	Investigational for formal risk-group assignment	[42,43,44,45]
L1CAM	NSMP/p53abn	Associated with aggressive features and adverse outcomes	Investigational prognostic refinement	[46,50]
Chromosome 1q alteration	NSMP	May identify an adverse NSMP subset	Investigational; requires assay and cutoff standardization	[28,41,50]
FGFR2	NSMP/MMRd-enriched	Potential target for FGFR-directed therapy	Clinical-trial/investigational biomarker	[63,64,65]
PIK3CA/PTEN	Endometrioid/NSMP	Defines PI3K-AKT-mTOR pathway activation	Investigational for treatment selection outside trials	[56,57,58,59,60,61,62]
ARID1A	MMRd/NSMP	May relate to DNA-repair vulnerability and immunogenicity	Investigational; prospective clinical utility not established	[47,48,49]

Abbreviations: ER, estrogen receptor; IHC, immunohistochemistry; ISH, in situ hybridization; MMRd, mismatch repair-deficient; MSI-H, microsatellite instability-high; NGS, next-generation sequencing; NSMP, no specific molecular profile; PR, progesterone receptor. “Established” and “clinically actionable” refer to defined guideline-supported or regulatory contexts, not universal use in every patient.

## Data Availability

No new data were created in this systematic review. Data availability is subject to the original studies included in the review.

## Supplementary Materials

The following supporting information can be downloaded at: https://www.mdpi.com/article/10.3390/diagnostics16182902/s1, Supplementary File S1: Complete Database Search Strategies. Supplementary File S2: the completed PRISMA-ScR checklist.

## Abbreviations

The following abbreviations are used in this manuscript:

AI	Artificial intelligence
cfDNA	Circulating free DNA
ctDNA	Circulating tumor DNA
DSS	Disease-specific survival
EC	Endometrial cancer
ER	Estrogen receptor
ESGO	European Society of Gynaecological Oncology
FIGO	International Federation of Gynecology and Obstetrics
FGFR2	Fibroblast growth factor receptor 2
HER2	Human epidermal growth factor receptor 2
IHC	Immunohistochemistry
ISH	In situ hybridization
LVSI	Lymphovascular space invasion
MMR	Mismatch repair
MMRd	Mismatch repair-deficient
MSI-H	Microsatellite instability-high
NCCN	National Comprehensive Cancer Network
NGS	Next-generation sequencing
NOS	Newcastle–Ottawa Scale
NSMP	No specific molecular profile
OS	Overall survival
PCR	Polymerase chain reaction
PFS	Progression-free survival
POLE	DNA polymerase epsilon
POLEmut	Pathogenic POLE-mutated molecular subgroup
PR	Progesterone receptor
PRISMA	Preferred Reporting Items for Systematic Reviews and Meta-Analyses
ProMisE	Proactive Molecular Risk Classifier for Endometrial Cancer
p53abn	p53-abnormal immunohistochemical pattern
QUADAS-2	Quality Assessment of Diagnostic Accuracy Studies-2
RFS	Recurrence-free survival
RoB 2	Revised Cochrane Risk of Bias tool
TCGA	The Cancer Genome Atlas
TP53mut	Sequencing-confirmed pathogenic TP53 alteration
