# Supplementary material for: Molecular Classification of Endometrial Cancer in the Era of Precision Oncology: Prognostic Significance, Emerging Biomarkers, and Personalized Therapeutic Strategies—A Systematic Review
Source: Diagnostics (Basel). 2026 Sep 9;16(18):2902. doi: 10.3390/diagnostics16182902 (PMC13605146; doi:10.3390/diagnostics16182902)
Supplement: Supplementary file 1 [file diagnostics-16-02902-s001.zip › diagnostics-4437142 PRISMA 2020 checklist.pdf]

PRISMA 2020 Checklist

Section and Topic	Item #	Checklist item	Location where item is reported
**TITLE**			
Title	1	Identify the report as a systematic review.	Title: “Molecular Classification of Endometrial Cancer in the Era of Precision Oncology: Prognostic Significance, Emerging Biomarkers, and Personalized Therapeutic Strategies—A Systematic Review.”
**ABSTRACT**			
Abstract	2	See the PRISMA 2020 for Abstracts checklist.	Abstract (structured Background/Objectives, Methods, Results, Conclusions).
**INTRODUCTION**			
Rationale	3	Describe the rationale for the review in the context of existing knowledge.	Section 1, Introduction.
Objectives	4	Provide an explicit statement of the objective(s) or question(s) the review addresses.	End of Section 1, Introduction: review aims stated explicitly.
**METHODS**			
Eligibility criteria	5	Specify the inclusion and exclusion criteria for the review and how studies were grouped for the syntheses.	Section 2.3, Eligibility Criteria (PICOS framework; inclusion and exclusion criteria); Section 2.7, Data Synthesis (grouping by molecular subgroup, biomarker, therapeutic context, and study design).
Information sources	6	Specify all databases, registers, websites, organisations, reference lists and other sources searched or consulted to identify studies. Specify the date when each source was last searched or consulted.	Section 2.2, Search Strategy: PubMed/MEDLINE, Embase, Scopus, Web of Science Core Collection, Cochrane Library; reference lists and trial registries/gray-literature sources also screened. Search period reported through 30 June 2026; database-specific last-search dates are not separately stated.
Search strategy	7	Present the full search strategies for all databases, registers and websites, including any filters and limits used.	Section 2.2, Search Strategy states that complete database-specific syntax, field tags, Boolean operators, date limits, and language limits are provided in the Supplementary Material.
Selection process	8	Specify the methods used to decide whether a study met the inclusion criteria of the review, including how many reviewers screened each record and each report retrieved, whether they worked independently, and if applicable, details of automation tools used in the process.	Section 2.4, Screening and Selection Process: two reviewers independently screened titles/abstracts and full texts; disagreements resolved by consensus with third senior reviewer adjudication. No automation tool is reported.
Data collection process	9	Specify the methods used to collect data from reports, including how many reviewers collected data from each report, whether they worked independently, any processes for obtaining or confirming data from study investigators, and if applicable, details of automation tools used in the process.	Section 2.5, Data Extraction: two investigators independently extracted data using a standardized form; discrepancies resolved by re-examining the source publication and consensus. Contact with study investigators and automation tools are not reported.
Data items	10a	List and define all outcomes for which data were sought. Specify whether all results that were compatible with each outcome domain in each study were sought (e.g. for all measures, time points, analyses), and if not, the methods used to decide which results to collect.	Section 2.5, Data Extraction: OS, DSS, PFS, RFS, hazard ratios, therapeutic interventions and other clinically relevant outcomes are listed. A separate rule for selecting among multiple compatible outcome results is not reported.
	10b	List and define all other variables for which data were sought (e.g. participant and intervention characteristics, funding sources). Describe any assumptions made about any missing or unclear information.	Section 2.5, Data Extraction: author, year, country, study design, sample size, histology, classifier/assays, POLE/MMR/MSI/p53/TP53 variables, molecular subgroup distribution, biomarkers and follow-up. Assumptions for missing/unclear information are not separately reported.

Section and Topic	Item #	Checklist item	Location where item is reported
Study risk of bias assessment	11	Specify the methods used to assess risk of bias in the included studies, including details of the tool(s) used, how many reviewers assessed each study and whether they worked independently, and if applicable, details of automation tools used in the process.	Section 2.6, Quality Appraisal and Risk of Bias Tools: two reviewers independently assessed risk of bias using QUADAS-2, Newcastle–Ottawa Scale, RoB 2, or structured narrative appraisal as appropriate; disagreements resolved by consensus. No automation tool is reported.
Effect measures	12	Specify for each outcome the effect measure(s) (e.g. risk ratio, mean difference) used in the synthesis or presentation of results.	Section 2.5 lists hazard ratios among extracted variables; Section 2.7 states that a quantitative meta-analysis was not performed. No single prespecified effect measure was used across the narrative synthesis.
Synthesis methods	13a	Describe the processes used to decide which studies were eligible for each synthesis (e.g. tabulating the study intervention characteristics and comparing against the planned groups for each synthesis (item #5)).	Sections 2.3 and 2.7: eligibility based on PICOS and studies interpreted/grouped according to study design, molecular subgroup, biomarker, therapeutic context, and clinical question.
	13b	Describe any methods required to prepare the data for presentation or synthesis, such as handling of missing summary statistics, or data conversions.	Not separately reported; no quantitative meta-analysis or data conversion procedure was described.
	13c	Describe any methods used to tabulate or visually display results of individual studies and syntheses.	Section 2.7, Data Synthesis; results presented narratively with Tables 1–4 and Figures 1–4.
	13d	Describe any methods used to synthesize results and provide a rationale for the choice(s). If meta-analysis was performed, describe the model(s), method(s) to identify the presence and extent of statistical heterogeneity, and software package(s) used.	Section 2.7, Data Synthesis: narrative synthesis was prespecified because of methodological heterogeneity in designs, assays, populations, treatment exposure, outcome definitions, and follow-up. Meta-analysis was not performed; therefore meta-analytic models/software are not applicable.
	13e	Describe any methods used to explore possible causes of heterogeneity among study results (e.g. subgroup analysis, meta-regression).	Section 2.7: differences were examined in relation to stage/histology, assay platform/variant classification, biomarker cutoffs, treatment protocols, follow-up duration, and multivariable adjustment.
	13f	Describe any sensitivity analyses conducted to assess robustness of the synthesized results.	Not applicable / not performed; no sensitivity analysis is reported.
Reporting bias assessment	14	Describe any methods used to assess risk of bias due to missing results in a synthesis (arising from reporting biases).	No formal reporting-bias assessment is described; quantitative meta-analysis was not performed.
Certainty assessment	15	Describe any methods used to assess certainty (or confidence) in the body of evidence for an outcome.	Sections 2.7 and 3.4: no formal GRADE assessment or single overall certainty rating was assigned; evidence was weighted qualitatively by study design, risk of bias, consistency, directness, and clinical applicability.
**RESULTS**			
Study selection	16a	Describe the results of the search and selection process, from the number of records identified in the search to the number of studies included in the review, ideally using a flow diagram.	Section 3.1, Study Selection, and Figure 1 (PRISMA 2020 flow diagram): 2843 records identified, 716 duplicates removed, 2127 screened, 183 full texts assessed, 74 primary studies included.
	16b	Cite studies that might appear to meet the inclusion criteria, but which were excluded, and explain why they were excluded.	Section 3.1 reports aggregate reasons and counts for 109 full-text exclusions. Individual potentially eligible excluded reports are not separately cited/listed.
Study	17	Cite each included study and present its characteristics.	Section 3.2 and Table 1 present characteristics of selected

Section and Topic	Item #	Checklist item	Location where item is reported
characteristics			representative primary studies; the manuscript states that 74 studies were included. A single table listing characteristics of all 74 studies is not provided in the main text.
Risk of bias in studies	18	Present assessments of risk of bias for each included study.	Section 3.4 and Table 3 provide a design-level summary of risk-of-bias patterns and recurrent concerns. Study-level risk-of-bias assessments for each of the 74 included studies are not separately tabulated.
Results of individual studies	19	For all outcomes, present, for each study: (a) summary statistics for each group (where appropriate) and (b) an effect estimate and its precision (e.g. confidence/credible interval), ideally using structured tables or plots.	Sections 3.2–3.5 provide narrative results and selected representative evidence in Tables 1, 2 and 4. Study-by-study summary statistics/effect estimates with precision for all 74 studies are not uniformly presented because the review used narrative synthesis.
Results of syntheses	20a	For each synthesis, briefly summarise the characteristics and risk of bias among contributing studies.	Sections 3.2–3.5 and Section 3.4; Table 3 summarizes risk-of-bias patterns by study design.
	20b	Present results of all statistical syntheses conducted. If meta-analysis was done, present for each the summary estimate and its precision (e.g. confidence/credible interval) and measures of statistical heterogeneity. If comparing groups, describe the direction of the effect.	Not applicable: no quantitative meta-analysis/statistical synthesis was performed. Narrative synthesis is reported in Sections 3.3–3.5.
	20c	Present results of all investigations of possible causes of heterogeneity among study results.	Sections 3.3, 4.1 and 4.2 discuss heterogeneity by stage, histology, assay platform, molecular context, treatment exposure, follow-up, and biomarker definitions.
	20d	Present results of all sensitivity analyses conducted to assess the robustness of the synthesized results.	Not applicable: no sensitivity analyses were performed/reported.
Reporting biases	21	Present assessments of risk of bias due to missing results (arising from reporting biases) for each synthesis assessed.	Not formally assessed/reported; no meta-analysis was performed.
Certainty of evidence	22	Present assessments of certainty (or confidence) in the body of evidence for each outcome assessed.	No formal GRADE/certainty assessment was performed. Section 3.4 and Section 4.6 describe qualitative weighting and limitations of the evidence.
**DISCUSSION**			
Discussion	23a	Provide a general interpretation of the results in the context of other evidence.	Section 4, Discussion, especially Sections 4.1–4.4.
	23b	Discuss any limitations of the evidence included in the review.	Sections 4.1, 4.2 and 4.6.
	23c	Discuss any limitations of the review processes used.	Section 4.6, Strengths and Limitations: no prospective protocol registration, English-language restriction, heterogeneity, no meaningful meta-analysis, no formal GRADE assessment, and limitations of recent/retrospective evidence.
	23d	Discuss implications of the results for practice, policy, and future research.	Sections 4.2–4.5 and Section 5, Research Priorities and Future Perspectives.
**OTHER INFORMATION**			
Registration and protocol	24a	Provide registration information for the review, including register name and registration number, or state that the review was not registered.	Section 2.1, Protocol and Registration: protocol was not prospectively registered in PROSPERO or another public registry.

PRISMA

PRISMA 2020 Checklist

Section and Topic	Item #	Checklist item	Location where item is reported
	24b	Indicate where the review protocol can be accessed, or state that a protocol was not prepared.	Section 2.1: an a priori protocol was developed; no public protocol location/access link is reported.
	24c	Describe and explain any amendments to information provided at registration or in the protocol.	No amendments to the a priori protocol are reported in the manuscript.
Support	25	Describe sources of financial or non-financial support for the review, and the role of the funders or sponsors in the review.	Funding statement: funded by LLP “National Research Oncology Center”, Astana, Kazakhstan; funding number not applicable. The funder’s role is not separately stated.
Competing interests	26	Declare any competing interests of review authors.	Conflicts of Interest statement: authors declare no conflicts of interest.
Availability of data, code and other materials	27	Report which of the following are publicly available and where they can be found: template data collection forms; data extracted from included studies; data used for all analyses; analytic code; any other materials used in the review.	Data Availability Statement: no new data were created; data availability is subject to the original included studies. Supplementary Materials are stated to include the completed PRISMA 2020 checklist and complete database-specific search strategies.

From: Page MJ, McKenzie JE, Bossuyt PM, Boutron I, Hoffmann TC, Mulrow CD, et al. The PRISMA 2020 statement: an updated guideline for reporting systematic reviews. BMJ 2021;372:n71. doi: 10.1136/bmj.n71. This work is licensed under CC BY 4.0. To view a copy of this license, visit https://creativecommons.org/licenses/by/4.0/
