# Supplementary material for: Molecular Classification of Endometrial Cancer in the Era of Precision Oncology: Prognostic Significance, Emerging Biomarkers, and Personalized Therapeutic Strategies—A Systematic Review
Source: Diagnostics (Basel). 2026 Sep 9;16(18):2902. doi: 10.3390/diagnostics16182902 (PMC13605146; doi:10.3390/diagnostics16182902)
Supplement: Supplementary file 1 [file diagnostics-16-02902-s001.zip › diagnostics-4437142-supplementary.pdf]

Supplementary File S1

Complete Database Search Strategies

Molecular Classification of Endometrial Cancer in the Era of Precision Oncology: Prognostic Significance, Emerging Biomarkers, and Personalized Therapeutic Strategies—A Systematic Review

Author verification required before submission

The database strings below operationalize the concepts reported in the revised Methods. Before resubmission, compare each platform name, exact string, final search date, and any applied limit with the saved database search histories. Amend this file wherever the archived executed strategy differs.

Search scope and general limits

- Population/topic: histologically confirmed endometrial carcinoma and TCGA-derived or clinically applicable molecular classification.
- Coverage period: 1 January 2013 through 30 June 2026.
- Language: English.
- Geographic restrictions: none.
- Eligible evidence: original primary research; guidelines and secondary syntheses were used only for contextual interpretation and reference-list screening.

S1. PubMed/MEDLINE

Platform/interface: PubMed

```
("Endometrial Neoplasms"[Mesh] OR "endometrial cancer"[tiab] OR "endometrial
carcinoma"[tiab])
AND ("The Cancer Genome Atlas"[tiab] OR TCGA[tiab] OR "molecular classification"[tiab] OR
"molecular subtype*"[tiab] OR ProMisE[tiab] OR TransPORTEC[tiab])
AND (POLE[tiab] OR "polymerase epsilon"[tiab] OR "mismatch repair"[tiab] OR MMRd[tiab] OR
dMMR[tiab] OR "microsatellite instability"[tiab] OR MSI[tiab] OR "MSI-H"[tiab] OR
p53[tiab] OR TP53[tiab] OR NSMP[tiab] OR "no specific molecular profile"[tiab])
AND (prognos*[tiab] OR survival[tiab] OR recurrence[tiab] OR biomarker*[tiab] OR
predict*[tiab] OR treatment[tiab] OR therap*[tiab])
AND ("2013/01/01"[Date - Publication] : "2026/06/30"[Date - Publication])
AND English[lang]
```

S2. Embase

Platform/interface: Embase.com syntax

Confirm the exact Embase interface used; field tags and limit syntax differ between Embase.com and Ovid Embase.

```
('endometrium tumor'/exp OR 'endometrial cancer':ti,ab,kw OR 'endometrial
carcinoma':ti,ab,kw)
AND ('cancer genome atlas':ti,ab,kw OR tcga:ti,ab,kw OR 'molecular
classification':ti,ab,kw OR 'molecular subtype*':ti,ab,kw OR promise:ti,ab,kw OR
transportec:ti,ab,kw)
AND (pole:ti,ab,kw OR 'dna polymerase epsilon':ti,ab,kw OR 'mismatch repair':ti,ab,kw OR
mmrd:ti,ab,kw OR dmmr:ti,ab,kw OR 'microsatellite instability':ti,ab,kw OR msi:ti,ab,kw
OR 'msi h':ti,ab,kw OR p53:ti,ab,kw OR tp53:ti,ab,kw OR nsmp:ti,ab,kw OR 'no specific
molecular profile':ti,ab,kw)
AND (prognos*:ti,ab,kw OR survival:ti,ab,kw OR recurrence:ti,ab,kw OR biomarker*:ti,ab,kw
OR predict*:ti,ab,kw OR treatment:ti,ab,kw OR therap*:ti,ab,kw)
AND [2013-2026]/py AND [english]/lim
```

S3. Scopus

Platform/interface: Elsevier Scopus

```
TITLE-ABS-KEY(("endometrial cancer" OR "endometrial carcinoma")
AND ("The Cancer Genome Atlas" OR TCGA OR "molecular classification" OR "molecular
subtype*" OR ProMisE OR TransPORTEC)
AND (POLE OR "polymerase epsilon" OR "mismatch repair" OR MMRd OR dMMR OR "microsatellite
instability" OR MSI OR "MSI-H" OR p53 OR TP53 OR NSMP OR "no specific molecular profile")
AND (prognos* OR survival OR recurrence OR biomarker* OR predict* OR treatment OR
therap*))
AND PUBYEAR > 2012 AND PUBYEAR < 2027
AND (LIMIT-TO(LANGUAGE, "English"))
```

S4. Web of Science Core Collection

Platform/interface: Clarivate Web of Science

```
TS=(("endometrial cancer" OR "endometrial carcinoma")
AND ("The Cancer Genome Atlas" OR TCGA OR "molecular classification" OR "molecular
subtype*" OR ProMisE OR TransPORTEC)
AND (POLE OR "polymerase epsilon" OR "mismatch repair" OR MMRd OR dMMR OR "microsatellite
instability" OR MSI OR "MSI-H" OR p53 OR TP53 OR NSMP OR "no specific molecular profile")
AND (prognos* OR survival OR recurrence OR biomarker* OR predict* OR treatment OR
therap*))
AND PY=(2013-2026) AND LA=(English)
```

S5. Cochrane Library

Platform/interface: Cochrane Library advanced search

```
#1 MeSH descriptor: [Endometrial Neoplasms] explode all trees
#2 ("endometrial cancer" OR "endometrial carcinoma"):ti,ab,kw
#3 ("The Cancer Genome Atlas" OR TCGA OR "molecular classification" OR "molecular
subtype*" OR ProMisE OR TransPORTEC):ti,ab,kw
#4 (POLE OR "polymerase epsilon" OR "mismatch repair" OR MMRd OR dMMR OR "microsatellite
instability" OR MSI OR "MSI-H" OR p53 OR TP53 OR NSMP OR "no specific molecular
profile"):ti,ab,kw
#5 (prognos* OR survival OR recurrence OR biomarker* OR predict* OR treatment OR
therap*):ti,ab,kw
#6 (#1 OR #2) AND #3 AND #4 AND #5
Limits: publication date 1 January 2013 to 30 June 2026; English language.
```

S6. Additional searching

Reference lists of eligible primary studies and relevant systematic reviews, consensus statements, and clinical guidelines were screened manually. Trial registries and other gray-literature sources were screened to identify potentially eligible full-text reports. Conference abstracts without a full-text publication and unpublished records were not included in the formal evidence synthesis.

S7. Search documentation to retain with the review record

- Saved search-history export or screenshot for each database.
- Exact date and time of the final search in each database.
- Number of records retrieved before deduplication by database.
- Citation export files and deduplication log.
- Documentation of any update search performed after 30 June 2026.
